# Incidence and risk factors for patellofemoral dislocation in adults with Charcot‐Marie‐Tooth disease: An observational study

**DOI:** 10.1002/pri.1996

**Published:** 2023-02-19

**Authors:** Enza Leone, Sally Davenport, Claire Robertson, Matilde Laurà, Mariola Skorupinska, Mary M. Reilly, Gita Ramdharry

**Affiliations:** ^1^ Physiotherapy Group UCL Great Ormond Street Institute of Child Health London UK; ^2^ Wimbledon Clinics Parkside Hospital London UK; ^3^ Department of Neuromuscular Diseases Queen Square Centre for Neuromuscular Diseases National Hospital for Neurology and Neurosurgery UCL Queen Square Institute of Neurology London UK

**Keywords:** Charcot‐Marie‐Tooth disease, incidence, patellofemoral dislocation, risk factors

## Abstract

**Background and Purpose:**

Patellofemoral (PF) dislocation is frequently encountered in clinical practice among people with Charcot‐Marie‐Tooth disease (CMT), but the frequency and risk factors for PF dislocation in adults with CMT are unknown. This study aimed to establish the incidence of PF dislocation in adults with CMT and to explore the risk factors associated with PF dislocation.

**Methods:**

This is a cross‐sectional study involving adults with a diagnosis of CMT, attending their outpatient clinics at a specialist neuromuscular centre in the United Kingdom. Eighty‐one individuals were interviewed about any PF dislocation and underwent a lower‐limb assessment, with a focussed knee examination, to identify possible risk factors for PF dislocation. The incidence of PF dislocation was expressed as a percentage (number of individuals with a positive history of patellar dislocation/overall sample) and the association between different risk factors and PF dislocation was explored using logistic regression analysis.

**Results:**

The incidence of PF dislocation was 22.2% (18/81). PF dislocation was associated with a younger age at the time of the assessment (*p* = 0.038) and earlier disease onset (*p* = 0.025). All people bar two who dislocated had CMT1A (88.9%), but there was no difference in terms of CMT distribution with the non‐dislocation group (*p* = 0.101). No association was found between PF dislocation and CMT severity measured by CMTSS (*p* = 0.379) and CMTES (*p* = 0.534). Patella alta (*p* = 0.0001), J‐sign (*p* = 0.004), lateral patellar glide (*p* = 0.0001), generalised joint hypermobility (*p* = 0.001) and knee flexors weakness (*p* = 0.008) were associated with an increased risk of dislocation. Patella alta (*p* = 0.010) and lateral patellar glide (*p* = 0.028) were independent PF dislocation predictors.

**Conclusions:**

PF dislocation was common in this cohort with CMT and was associated with multiple risk factors. Future studies should be conducted to confirm the present findings so that the identified risk factors may be addressed by clinicians through preventive, supportive and corrective measures.

## 1 INTRODUCTION

Charcot‐Marie‐Tooth disease (CMT) encompasses a genetically heterogeneous group of hereditary neuropathies with both motor and sensory manifestations (Reilly et al., 2011). It is the most frequent inherited neuropathy (prevalence of 1:2500) and CMT type 1A is the most common type (40%–50% of cases) (Barreto et al., 2016; Fridman et al., 2015). The hallmark feature of CMT is a gradual and distal progression of wasting, weakness and sensory loss, which affects firstly the intrinsic foot muscles, before involving the lower leg muscles and hands (Vinci & Perelli, 2002). Resultant muscle imbalance can lead to foot deformities including pes cavus (Holmes & Hansen, 1993). Altered foot posture and consequent misalignment then leads to an abnormal posture in stance and an altered gait (Newman et al., 2007; Vinci & Perelli, 2002).

The term patellofemoral (PF) dislocation is used to refer to the complete loss of contact between the patellofemoral joint surfaces (Diederichs et al., 2010). In the general population, the incidence of PF dislocation varies between 2.29/100.000 and 74.4/100.000 (Sillanpää et al., 2008; Waterman et al., 2011) and PF dislocation is associated with risk factors including trochlear dysplasia, patella alta, abnormal tibial tuberosity‐trochlear groove distance, and connective tissue disorders (Balcarek et al., 2010; Charles et al., 2013; Dejour et al., 1994; Köhlitz et al., 2013; Malfait et al., 2017; Nomura et al., 2006; Osman & Ebrahim, 2016). Clinicians noticed that there appeared to be a high frequency of PF dislocation among people with CMT. To date, only a single study has investigated the incidence of PF dislocation in people with CMT (14%) involving children with CMT1A (Main et al., 2012). The frequency with which patellofemoral dislocation occurs among adults with CMT and characteristics that predispose their patellofemoral joints (PFJ) to dislocation are still unknown. However, a number of those predisposing factors associated with patellofemoral instability seem to be intrinsically associated with the typical CMT characteristics. For example, generalised lower‐limb weakness may lead to a secondary impairment of the knee tendons and ligaments. As a consequence, joint laxity may be altered, resulting in patella dislocation due to an insufficient control of its movements (Carson et al., 1984). Similarly, muscle weakness affecting the quadriceps muscles (Greiwe et al., 2010), may facilitate the actions of the external forces acting to displace the patella, particularly weakness of the vastus medialis oblique that normally resists lateral patellar displacement. Additionally, foot deformities, observed in 74% of people with CMT (Laurá et al., 2018), may affect lower‐limb biomechanics (Lufler et al., 2017), thereby altering the load distribution across the knee joint (Barton et al., 2010). This will affect the structural integrity of the knee, which can result in PF instability. The consolidated rotational deformities of the distal femur and tibial torsion observed in CMT may expose people with this condition to an increased risk of PF dislocation, as they contribute to patella instability by respectively increasing patellofemoral contact pressure and laterally moving the kneecap during quadriceps contraction (Sillanpää et al., 2008). Considering that a number of the known risk factors for this dysfunction potentially align to the presentation of those with CMT, there is a need to investigate the incidence and risk factors for PF dislocation in adults with CMT. A better understanding of this phenomenon and knowledge of the risk factors would provide clinicians with relevant information for the avoidance and rehabilitation of PF dislocation in adults with CMT.

This study was undertaken to determine the incidence and the risk factors for PF dislocation in a cohort of adults with CMT attending a neuromuscular centre in the United Kingdom.

## 2 METHODS

A cross‐sectional observational study was conducted among adults with CMT who attended outpatient clinics at the Medical Research Council (MRC) Queen Square Centre for Neuromuscular diseases, National Hospital for Neurology and Neurosurgery, London. We recruited from 42 clinics between May 2019 and December 2019, with an average of five patients per clinic. Only people aged 18 and over with a clinically and/or genetically confirmed diagnosis of CMT were invited to participate. Patients with other concomitant conditions such as movement disorders and previous history of knee replacement were excluded. This study was approved by the Health and Social Care Research Ethics Committee A (HSC REC A) under the REC reference number 19/NI/0031. This study was also reviewed and given approval by the research department at UCL Great Ormond Street Institute of Child Health. Written informed consent was obtained from all participants at enrolment.

### 2.1 Patellofemoral assessment

Study participants were evaluated for PF instability by two assessors (EL and GR). The term PF dislocation was adopted to refer to complete dislocations, which occurred in either the presence or absence of any previous PF displacement. Information was recorded regarding any history of dislocation, number of dislocations, the mechanisms of injury and subsequent surgeries. People were asked whether they were apprehensive of recurrent dislocation. Disease severity was measured using the CMT Symptom Score (CMTSS) and CMT Examination Score (CMTES) (Murphy et al., 2011).

A physical examination of the knee joint was performed to check for signs of patellar instability typically associated with a higher risk of patellofemoral dislocation, such as patella alta, lateralisation of the tibial tuberosity, lateral patellar glide and J‐sign. While lateral patellar glide indicates a patellar displacement when medial and lateral forces are applied to the patella (Hryvniak et al., 2014), J‐sign refers to the inverted J‐path the patella takes, when knee flexion starts from a fully extended position, in order to engage the femoral groove (Hadidi et al., 2018).

The assessment also included an evaluation of the lower limb muscle strength, foot posture, screening for generalised joint hypermobility (GJH) and connective tissue disorders (Ehlers‐Danlos syndrome [EDS]). These measures were undertaken using: MRC scale, Foot Posture Index (FPI), Beighton score and the Diagnostic criteria for EDS score, respectively. The age‐dependent cut‐off points for GJH were ≥5/9 for people aged under 50 and ≥4/9 for people >50 years (Malfait et al., 2017). The FPI raw data were converted into Rasch transformed numerical scores in line with Keenan et al. (2007) (Keenan et al., 2007). The lower limb assessment comprised a standard neurological assessment of sensory impairment, including joint position sense (JPS), vibration sense and pinprick (Prabhakar et al., 2019).

### 2.2 Minimal sample size calculation

The minimal sample size was calculated based on the number of variables (e.g., patella alta, lateral patellar glide, lateralised tibial tuberosity, GJH and EDS) to include in the logistic regression model (*n* = 6). A minimum of 10 observations per variable is recommended for logistic regression indicating that a sample size of 60 is required for six variables (Peduzzi et al., 1996). To allow for any incomplete data or withdrawal during the data collection, a minimal sample of 80 participants was calculated.

### 2.3 Statistical analysis

Statistical analysis was conducted using Statistical Package for Social Science (SPSS) software (IBM SPSS Statistics Version 25). The data was tested for normality using graphical methods such as histograms and Shapiro‐Wilk test where *p*‐values greater than 0.05 were referred to as significant (Bursac et al., 2008). For baseline demographics, continuous data was summarised as mean and standard deviation (SD) if normally distributed and as median and interquartile ranges (IQR) when non‐normally distributed. All categorical variables were presented as percentages. The Mann‐Whitney test was used to compare demographic characteristics of individuals with and without a positive history of PF dislocation. Characteristics of the two groups were also compared by using Chi‐square test and Fisher's exact test for normally and non‐normally distributed categorical variables, respectively.

Following the study by Main and colleagues (Main et al., 2012) exploring the incidence of PF in a child cohort with CMT, we calculated the incidence of PF dislocation as the number of individuals with a positive history of patellar dislocation divided by the overall sample and was expressed as a percentage. Comparative analysis of the risk factors for PF dislocation was conducted within the dislocation group. The association between PF dislocation and the potential predisposing factors was analysed by using Chi‐square test and Fisher's exact test for normally and non‐normally distributed categorical data, respectively. The Mann‐Whitney *U*‐test was used to compare non‐normally distributed continuous data in knees with and without a positive history of PF dislocation. The threshold for statistical significance was set at *p* < 0.05.

Logistic regression analysis was undertaken to identify the association between the outcome and predictors after adjusting for potential confounders. In a binary logistic regression scenario, the dependent binary outcome was the PF dislocation occurrence, while the predictors were the risk factors potentially associated with patellar displacement. Logistic regression analysis generated adjusted Odds Ratio (aOR) with 95% CIs.

## 3 RESULTS

### 3.1 Study population

From the 42 clinics (a total of 180 patients), 81 individuals both met the inclusion criteria and agreed to be enroled in the study. The study population consisted of 38 (46.9%) males and 43 (53.1%) females with a median age of 43 years (IQR = 29.5–54.0). The median age at disease onset was 12 years (IQR = 4.0–19.0 years) and CMT1A was the most prevalent subtype among participants (53.1%) (Table 1).

**TABLE 1:** Demographics of the study participants.

Characteristics	CMT total sample (*n* = 81)	Non‐dislocation group (*n* = 63)	Dislocation group (*n* = 18)
Age (years), median (IQR)	43 (25)	46 (26)	30.5 (22)*
Sex, *n* (%)			
Male	38 (49.6%)	32 (50.8%)	6 (33.3%)
Female	43 (53.1%)	31 (49.2%)	12 (66.7%)
CMT subtype, *n* (%)			
CMT 1A	43 (53.1%)	27 (42.9%)	16 (88.9%)
CMT 1B	1 (1.2%)	3 (4.8%)	0 (0.0%)
CMT 1F	1 (1.2%)	1 (1.6%)	0 (0.0%)
CMT 1X	10 (12.3%)	9(14.3%)	1 (5.6%)
CMT 2	8 (9.9%)	8 (12.7%)	0 (0.0%)
CMT 2 dHMN	9 (11.1%)	9 (14.3%)	0 (0.0%)
CMT 2A	2 (2.5%)	2 (3.2%)	0 (0.0%)
I‐CMT	4 (4.9%)	3 (4.8%)	1 (5.6%)
CMT 4C	1 (1.2%)	1 (1.6%)	0 (0.0%)
CMT severity			
CMTSS	3.9 ± 2.0	3.8 ± 2.0	4.4 ± 1.7***
CMTES	10.1 ± 4.1	10.0 ± 4.2	10.6 ± 3.7****
Age at onset (years), median (IQR)	12 (15)	13 (20)	6 (9)**

Abbreviations: CMT, Charcot‐Marie‐Tooth disease; CMT 2 dHMN, CMT 2 distal hereditary motor neuropathy; CMTES, CMT Examination Score; CMTSS, CMT Symptom Score; I‐CMT, CMT intermediate; IQR, interquartile range.

*p = 0.038, **p = 0.025, ***p = 0.379, ****p = 0.534.

### 3.2 Incidence of patellofemoral dislocation

The incidence of PF dislocation was 22.2% (18/81). Recurrent dislocations were experienced by most of the dislocation group (66.7%, 12/18) and the median number of dislocations per person was 3.5 (IQR = 1.0–8.0). Patellar dislocation was bilateral in 33.3% (6/18). The median age of the participants at first dislocation was 16.5 years (IQR = 13.8–18.5 years).

### 3.3 Group comparison

Study participants were stratified in two subgroups defined according to their history of dislocation (Table 1). The dislocation group had a median age of 30.5 years (IQR = 26.8–49.0 years) and was significantly younger than the non‐dislocation group (Median = 45.0 years, IQR = 32.0–58.0 years), *U* = 384.00, *p* = 0.038. Most of the individuals with a positive history of dislocation were women (*n* = 12, 66.7%), but the two groups did not significantly differ in terms of sex distribution, *X* ^*2*^(1, *N* = 81) = 1.71, *p* = 0.190. There was no difference in disease severity between the two groups (CMTSS: *p* = 0.379; CMTES: *p* = 0.534). Compared to the non‐dislocation group, there was a considerably higher proportion of individuals with CMT1A in the dislocation group (88.9% vs. 42.9%); however, this difference did not achieve statistical significance (Fisher's exact test, *p* = 0.101). The dislocation group was significantly younger (Median = 6 years, IQR = 2.75–12.00) at disease onset than the non‐dislocation group (Median = 13 years, IQR = 5.0–25.00), *U* = 369.50, *p* = 0.025.

### 3.4 Nature of patellofemoral dislocation

**FIGURE 1:**
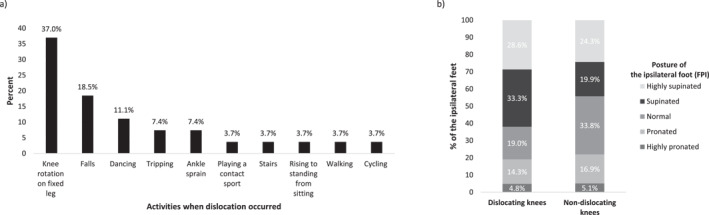
(a) Circumstances of patellar dislocation; (b) Posture of the feet ipsilateral to knees with and without a positive history of patellofemoral dislocation.

Most of the dislocations (37.0%) resulted from a knee rotation around a vertical axis, while the foot remained placed on the ground. Other less common scenarios were falling, dancing, tripping and an episode of ankle sprain (Figure 1a). Following patellar dislocation, 33.3% (6/18) of the dislocation group underwent surgical interventions.

### 3.5 Fear of PF dislocation

Eleven participants (61.1%, 11/18) said they were fearful of dislocating their patella again. Of them, 72.7% (8/11) had a history of recurrent dislocation.

### 3.6 Risk factors for patellar dislocation

#### 3.6.1 Anatomic risk factors

Within the dislocation group, there was a significantly higher proportion of knees with patella alta, lateral patellar glide and J‐sign as compared to those without these characteristics (*p* = 0.0001, *p* = 0.0001 and *p* = 0.004, respectively) (Table 2). The OR for the association between PF dislocation and patella alta, lateral patellar glide and J‐sign was respectively large at 8.7 (95% CI: 3.3–22.8), 15.0 (95% CI: 3.4–66.3) and 4.2 (95% CI: 1.5–11.9) indicating a strong relationship between these risk factors and PF displacement. No statistically significant association was found between lateralised tibial tuberosity and PF dislocation, *X* ^*2*^(1, *N* = 161) = 0.177, *p* = 0.674.

**TABLE 2:** Risk factors for patellofemoral dislocation.

Risk factor (exposure)	*N* (knees)	*n* (%) dislocating knees in non‐exposed group	*n* (%) dislocating knees in exposed group	Univariate analysis		*p V*alue	Multivariate analysis		*p Value*
				OR	95% CI		aOR	95% CI	
Patella alta	161	7 (6.1%)	17 (36.2%)	8.7	3.3–22.8	0.0001*	**4.6**	**1.4**– **14.6**	**0.010**
Lateral patellar glide	161	2 (2.5%)	22 (27.5%)	15.0	3.4–66.3	0.0001*	**6.0**	**1.2**–**29.4**	**0.028**
J‐sign	161	5 (6.5%)	19 (22.6%)	4.2	1.5–11.9	0.004*	1.3	0.4–4.5	0.686
Lateralised tibial tuberosity	161	5 (12.8%)	19 (15.6%)	1.3	0.4–3.6	0.674*	0.9	0.2–3.6	0.869
GJH (Beighton score)	156	13 (9.7%)	9 (40.9%)	6.4	2.3–18.0	0.001*	2.1	0.6–7.0	0.213
EDS	156	19 (12.8%)	4 (50.0%)	6.8	1.6–29.5	0.017**	4.4	0.6–29.8	0.124

Risk factor	*N* (Knees)	Non‐dislocating knees (*n* = 121)	Dislocating knees (*n* = 19)			*p* Value			
Knee flexors strength	162	Non‐dislocating knees (*n* = 138)	Dislocating knees (*n* = 24)			0.008**	0.6	0.2–2.5	0.522
4		6 (4.3%)	0 (0.0%)						
4+		4 (2.9%)	5 (20.8%)						
5		128 (92.8%)	19 (79.2%)						

*Note*: **Bold** values denote statistical significance at the *p* < 0.05 level.

Abbreviations: aOR, adjusted odds ratio; CI, confidence interval; EDS, Ehlers‐Danlos syndromes; GJH, generalised joint hypermobility; OR, odds ratio.

*Chi‐squared. ** Fisher’s exact test. *** Mann‐Whitney U‐test.

### 3.7 Other risk factors

#### 3.7.1 Muscle weakness

Dislocating and non‐dislocating knees were compared in terms of the muscle strength of nine muscle groups of the ipsilateral lower limb (supplementary file 1). The strength of the knee flexors ipsilateral to dislocating knees was scored as normal significantly less frequently than that of those ipsilateral to non‐dislocating knees (*p* = 0.008).

#### 3.7.2 Sensation

There were no significant differences between dislocating and non‐dislocating knees in terms of the level of loss of vibration (*p* = 0.840), pinprick (*p* = 0.625) and JPS (*p* = 0.885) of their ipsilateral leg (supplementary file 2).

#### 3.7.3 Foot posture

The posture of the ipsilateral foot was examined for dislocating and non‐dislocating knees. The dislocating knee group presented with median FPI score lower than that of the non‐dislocating group. Although this difference did not reach statistical significance (dislocation median = −0.91, IQR = 5.21, non‐dislocation median = 0.5, IQR = 5.65, *U* = 1193.50, *p* = 0.226), the lower FPI values observed in the dislocation group indicated a trend towards a supinated foot posture on the same side as the knees that had dislocated. According to their score on the FPI scale, feet ipsilateral to dislocating and non‐dislocating knees were classified into five categories of foot posture (Figure 1b). While most feet ipsilateral to a dislocating knee were categorised as supinated (33.3%), normal foot posture was the most common category in the non‐dislocation group (33.8%). No significant difference was found between dislocating (38.1%) and non‐dislocating knees (29.2%) in terms of the frequency of surgery on their ipsilateral foot, *X* ^*2*^(1, *N* = 158) = 0.68, *p* = 0.409.

#### 3.7.4 Generalised joint hypermobility (GJH) and Ehlers–Danlos syndromes (EDS)

Within the group of knees with a prior history of PF dislocation, there was a significantly higher proportion of knees belonging to individuals with signs of GJH compared to those belonging to people who did not have GJH, *X* ^*2*^(1, *N* = 156) = 10.795, *p* = 0.001 (Table 2). Additionally, the dislocation group had a significantly higher percentage of knees belonging to people who scored above the threshold of the EDS scale than those who did not (Fisher's exact test *p* = 0.017).

### 3.8 Logistic regression analysis

Only patella alta and lateral patellar glide were identified as independent predictors of PF dislocation (*p* = 0.010 and *p* = 0.028, respectively) (Table 2). J‐sign, GJH, EDS and knee flexors weakness were not independently associated with PF dislocation after adjusting for the other confounding factors included in the model. The Hosmer and Lemeshow ‘goodness‐of‐fit test’ showed a high significance of the model, with a chi‐square of 3.307 and *p* = 0.0001.

## 4 DISCUSSION

This was the first study to investigate the incidence and risk factors for PF dislocation in a cohort of adults with CMT. A total of 18 people out of the 81 reported a positive history of PF dislocation equating to an incidence of 22.2%. The incidence of PF dislocation in this study involving adults was higher than the reported incidence in children with CMT (14%) (Main et al., 2012). This may suggest that people with CMT continue to experience PF dislocation into adulthood. People with CMT experienced their first‐time dislocation in their second decade of life (median age of 16.5 years). This finding is in line with the study by Fithian et al. (2004) (Fithian et al., 2004) involving a non‐CMT population and may suggest that the possible association of skeletal immaturity and a relatively higher level of physical activity seen in children with CMT compared to adults may have rendered people more prone to PF dislocation during adolescence. Because of different methods employed across the studies to estimate the incidence of this phenomenon, the incidence in adults with CMT is not directly comparable with that reported by other studies involving non‐CMT population (SILLANPÄÄ et al., 2008; Waterman et al., 2011).

All people bar two in the dislocation group had CMT1A (88.9%, 16/18). The dislocation group developed symptoms of CMT at a significantly earlier age than the non‐dislocation group (*p* = 0.038). Interestingly, people with CMT1A who dislocated were also significantly younger at point of diagnosis than those with CMT1A who did not (5 vs. 8 years old) (*p* = 0.040). This may suggest that a more normal physical development in very early childhood may protect people with CMT from later PF dislocation. Furthermore, although CMT1A was not statistically associated with PF dislocation, there was a compelling trend and a larger sample may be required to accurately ascertain if this is a risk factor.

Knee rotation on a fixed leg was reported as the primary cause of PF dislocation (37% of cases). A non‐contact twisting or pivoting injury to the knee was also found to be the most frequent mechanism of injury related to PF dislocation in the musculoskeletal literature (Parikh et al., 2018). However, Parikh et al. (2018) reported that these types of injury occurred often during sports and high‐risk pivoting activities whereas PF dislocations appeared to be more common in people with CMT after low‐risk pivoting tasks (Parikh et al., 2018). This could be because individuals with CMT are a less physically active group (Ramdharry et al., 2017) and it may also reflect the possible anatomic abnormalities of the PFJ in the CMT population, as has been observed in the feet and hips (Bamford et al., 2009; SILLANPÄÄ et al., 2008).

Fear of experiencing future PF dislocation was common among people with CMT (61.1%). This may suggest that PF dislocation may have affected the quality of life of adults with CMT, especially that of those sustaining recurrent dislocations as 72.7% of those fearful of dislocating again had a history of recurrent dislocations. Exploring this aspect further was beyond the scope of this study, but future studies should investigate the impact of PF dislocation on adults with CMT.

Patella alta, lateral patellar glide and J‐sign were strongly associated with an increased risk of experiencing PF dislocation in people with CMT. This in line with the musculoskeletal literature although more precise radiographical techniques to detect the presence of these predisposing characteristics were used in musculoskeletal studies (Balcarek et al., 2010; Shirley et al., 2012). This may indicate that a clinical assessment is a meaningful tool for screening potential cases of PF instability and that it may be an alternative to imaging techniques in clinical settings. Lateralised tibial tuberosity was not found to be a risk factor for PF dislocation in people with CMT. This may be because tibial torsion is frequently observed in adults with CMT (Newman et al., 2007).

PF dislocation was associated with hamstrings weakness in people with CMT (*p* = 0.008). Weak hamstrings could leave the knee vulnerable to tibiofemoral posterior translation and external rotation, leading to PF malalignment and elevated PF pressure. Hamstring strengthening is an advisable rehabilitation option with this presentation.

The knees of hypermobile individuals with CMT had significantly higher odds of experiencing PF dislocation. Similarly, having EDS scoring above the diagnostic screening threshold of the EDS scale was associated with a significantly higher risk of experiencing PF dislocation. These results are in line with other studies conducted in musculoskeletal populations (Nathan et al., 2018; Shirley et al., 2012) and may be because hypermobile joints are more unstable with increased range of movement, so more prone to dislocation.

Dislocating and non‐dislocating knees did not differ in terms of the posture of the ipsilateral foot, as measured by the FPI. Although not significant, there was a trend towards more supinated feet where knees dislocated. This may suggest that a supinated foot structure may lead to knee mechanical malalignment and uneven distribution of forces across the knee (Guichet et al., 2003), which may favour PF dislocations in people with CMT. This may also explain the higher prevalence of PF dislocation observed in CMT1A, as foot deformities, in particular pes cavus, are more frequently observed in CMT1A compared to other CMT subtypes (Laurá et al., 2018). Therefore, a larger sample is required to understand if this is truly a null result. If further investigation supports this as a real finding, conservative and surgical treatments to cavus feet could contribute to greater PF joint stability (Maynou et al., 2017).

Only patella alta and lateral patellar glide were independently associated with the increased risk of experiencing PF dislocation. The individual association between J‐sign, GJH, EDS, weak hamstrings and PF dislocation was attenuated after adjusting for the other factors in the model. This suggests that J‐sign, GJH, EDS, weak hamstrings did not have an independent role in determining the occurrence of PF dislocation. The heterogeneity of the group and smaller number of individuals who had dislocated will have underpowered this analysis, so further investigation in a larger group is required.

This study has its limitations. Firstly, the observational nature of this study did not allow us to determine any causal relationships between the risk factors and patellar dislocation. The findings of this study may have been subject to observer bias as the examiners were not blinded to the patient's prior history of dislocation due to the nature of the study. There may have been a sampling bias that could impact the incidence statistics. Patients with a history of knee problems may have been more likely to agree to participate. Another limitation of the study is the lack of a healthy age‐matched control group which may have underpowered the statistical analysis. Although the demographic characteristics of the sample were representative of the characteristics of the CMT population, future studies with larger sample sizes are needed to determine whether our study results generalise to the larger CMT population.

This study has several strengths. We prospectively assessed the incidence and the risk factors for PF dislocation using broad inclusion criteria. This was the first study conducted in the adult population with CMT to suggest possible knee morphological abnormalities which are unexplored as a presenting feature compared to the hip and foot. Additionally, study participants were assessed for PF instability through a cost‐effectiveness screening test, which is a reproducible tool adoptable in clinical practice.

To date, this is the first study investigating the incidence and the risk factors for PF dislocation in a cohort of adults with CMT. PF dislocation was common among adults with CMT (22.2%) and was predominantly experienced by young female adults with CMT1A. It was associated with multiple risk factors including patella alta, J‐sign, lateral patellar glide, hamstrings weakness, GJH and EDS. Patella alta and lateral patellar glide were also independently associated with an increased PF dislocation risk. Given the extent of the phenomenon, future studies with larger sample sizes are needed to further investigate this topic. Should the results of our study be confirmed by future research, colleagues may include consideration of patellar dislocation in their assessments of people with CMT, as there are a number of rehabilitation options available that can help manage this potentially debilitating presentation.

## 5 IMPLICATIONS ON PHYSIOTHERAPY PRACTICE

The findings of this study revealed that PF dislocation is predominantly experienced by young female adults with CMT1A and patella alta, J‐sign, lateral patellar glide, hamstrings weakness, GJH and EDS are all associated with a higher risk of PF dislocation. Thus, the examination of the PFJ is encouraged in clinical practice, especially in the assessment of young women with CMT1A. This may allow the early identification of risk factors for PF dislocation and the introduction of physiotherapy and rehabilitation programs, which can help manage PFJ instability and prevent PF dislocation.

## CONFLICTS OF INTEREST STATEMENT

All the authors have no other funding disclosures or conflicts of interest to declare.

## ETHICS STATEMENT

This study was approved by the Health and Social Care Research Ethics Committee A (HSC REC A) under the REC reference number: 19/NI/0031 and the Health Research Authority. This study was also reviewed and given approval by the research department at UCL Great Ormond Street Institute of Child Health.

## PATIENT CONSENT STATEMENT

All participants signed an informed consent form.

## Supporting information

Supporting Information S1

Supporting Information S2

## Data Availability

The data that support the findings of this study are available from the corresponding author upon reasonable request.
