# Supplementary material for: Incidence and risk factors for patellofemoral dislocation in adults with Charcot‐Marie‐Tooth disease: An observational study
Source: Physiother Res Int. 2023 Feb 19;28(3):e1996. doi: 10.1002/pri.1996 (PMC10909428; doi:10.1002/pri.1996)
Supplement: Supplementary file 1 — Supporting Information S1 [file PRI-28-e1996-s002.docx]

**Supplementary file 1. Manual muscle testing of nine muscle groups in dislocation and non-dislocation groups**

| Variables | Non-dislocation group | Dislocation group | Test statistic | *p* value |
| --- | --- | --- | --- | --- |
| Hip flexors (n=162) | (n=138) | (n=24) |  |  |
| 4  4+  5 | 4(2.9%)  4(2.9%)  130(94.2%) | 1(4.2%)  0(0.0%)  23(95.8%) | FET=0.652 | 0.773 |
| Hip extensors (n=162) | (n=138) | (n=24) |  |  |
| 2  3  4-  4  4+  5 | 1(0.7%)  1(0.7%)  1(0.7%)  1(0.7%)  5(3.6%)  129(93.5%) | 0(0.0%)  0(0.0%)  0(0.0%)  0(0.0%)  0(0.0%)  24(100.0%) | FET=2.574 | 1.000 |
| Hip abductors (n=160) | (n=136) | (n=24) |  |  |
| 3  4+  5 | 1(0.7%)  12(8.8%)  123(90.4%) | 0(0.0%)  0(0.0%)  24(100.0%) | FET=2.451 | 0.333 |
| Knee flexors (n=162) | (n=138) | (n=24) |  |  |
| 4  4+  5 | 6(4.3%)  4(2.9%)  128(92.8%) | 0(0.0%)  5(20.8%)  19(79.2%) | FET=9.424 | **0.008** |
| Knee extensors (n=162) | (n=127) | (n=24) |  |  |
| 4-  4  4+  5 | 1(0.7%)  3(2.2%)  7(5.1%)  127(92.0%) | 0(0.0%)  0(0.0%)  0(0.0%)  24(100.0%) | FET=1.373 | 0.789 |
| Ankle dorsiflexion (n=161) | (n=45) | (n=15) |  |  |
| 0  1  2  3  4-  4  4+  5 | 29(21.0%)  10(7.2%)  11(8.0%)  8(5.8%)  8(5.8%)  30(21.7%)  35(25.4%)  7(5.1%) | 4(17.4%)  0(0.0%)  1(4.3%)  3(13.0%)  5(21.7%)  5(21.7%)  4(17.4%)  1(4.3%) | FET=8.687 | 0.212 |
| Ankle plantarflexion(n=160) | (n=137) | (n=23) |  |  |
| 0  1  2  3  4-  4  4+  5 | 17(12.4%)  9(6.6%)  6(4.4%)  3(2.2%)  1(0.7%)  11(8.0%)  16(11.7%)  74(54.0%) | 3(13.3%)  1(4.3%)  0(0.0%)  0(0.0%)  1(4.3%)  4(17.4%)  2(8.7%)  12(52.2%) | FET=4.973 | 0.618 |
| Inversion (n=150) | (n=132) | (n=18) |  |  |
| 0  1  2  3  4-  4  4+  5 | 25(18.9%)  8(6.1%)  1(0.8%)  2(1.5%)  4(3.0%)  6(4.5%)  10(7.6%)  76(57.6%) | 6(33.3%)  0(0.0%)  0(0.0%)  1(5.6%)  0(0.0%)  0(0.0%)  1(5.6%)  10(55.6%) | FET=4.905 | 0.607 |
| Eversion (n=150) | (n=132) | (n=18) |  |  |
| 0  1  2  3  4-  4  4+  5 | 40(30.3%)  7(5.3%)  6(4.5%)  5(3.8%)  7(5.3%)  32(24.2%)  11(8.3%)  24(1.2%) | 6(33.3%)  2(11.1%)  2(11.1%)  1(5.6%)  0(0.0%)  4(22.2%)  1(5.6%)  2(11.1%) | FET=4.155 | 0.740 |
| Abbreviations: FET: Fisher’s exact test. **Bold** values denote statistical significance at the p<0.05 level. | | | | |
