# Supplementary material for: Incidence and risk factors for patellofemoral dislocation in adults with Charcot‐Marie‐Tooth disease: An observational study
Source: Physiother Res Int. 2023 Feb 19;28(3):e1996. doi: 10.1002/pri.1996 (PMC10909428; doi:10.1002/pri.1996)
Supplement: Supplementary file 2 — Supporting Information S2 [file PRI-28-e1996-s001.docx]

**Supplementary file 2. Sensory assessment in non-dislocation and dislocation group**

| Variables | Non-dislocation group | Dislocation group | Test statistic | *p* value |
| --- | --- | --- | --- | --- |
| Vibration sense (n=162) | (n=138) | (n=24) |  |  |
| Normal  Decreased at the foot  Decreased at the ankle  Decreased at the knee  Decreased at the ASIS  Decreased at costal margins | 33(23.9%)  2(1.4%)  66(47.8%)  26(18.8%)  5(3.6%)  6(4.3%) | 8(33.3%)  0(0.0%)  10(41.7%)  5(20.8%)  1(4.2%)  0(0.0%) | FET=1.954 | 0.840 |
| Pinprick sense (n=162) | (n=138) | (n=24) |  |  |
| Normal  Decreased at the foot  Decreased at the ankle  Decreased at the knee  Decreased at the ASIS | 35(25.4%)  9(6.5%)  72(52.2%)  20(14.5%)  2(1.4%) | 4(16.7%)  0(0.0%)  16(66.7%)  4(16.7%)  0(0.0%) | FET=2.651 | 0.625 |
| JPS (n=162) | (n=138) | (n=24) |  |  |
| Normal  Decreased at the foot  Decreased at the ankle  Decreased at the knee | 116(84.1%)  4(2.9%)  17(12.3%)  1(0.7%) | 22(91.7%)  0(0.0%)  2(8.3%)  0(0.0%) | FET=0.972 | 0.885 |
| Abbreviations: FET, Fisher’s exact test; ASIS, Anterior Superior lilac Spine; JNP, Joint Position Sense. | | | | |
